# Is adductor pollicis muscle thickness a good predictor of lean mass in adults?

**DOI:** 10.1016/j.clnu.2015.07.022

**Published:** 2016-10

**Authors:** Renata Moraes Bielemann, Bernardo Lessa Horta, Silvana Paiva Orlandi, Thiago Gonzalez Barbosa-Silva, Maria Cristina Gonzalez, Maria Cecília Assunção, Denise Petrucci Gigante

**Affiliations:** a Post-Graduate Program in Epidemiology, Federal University of Pelotas, Brazil; b Nutrition Department, Federal University of Pelotas, Brazil; c Post-Graduate Program in Health and Behavior, Catholic University of Pelotas, Brazil

**Keywords:** Anthropometry, Adductor pollicis muscle, Lean mass, Adults

## Abstract

**Background & aims:**

Lean mass (LM) is an important parameter in clinical outcomes, which highlights the necessity of reliable tools for its estimation. The adductor pollicis muscle thickness (APMT) is easily accessible and suffers minimal interference from the adjacent subcutaneous fat tissue.

**Objective:**

To assess the relationship between the APMT and LM in a sample of Southern Brazilian adults.

**Methods:**

Participants were adults from the 1982 Pelotas (Brazil) Birth Cohort. LM was measured by dual energy X-ray absorptiometry (DXA). LM and lean mass index (LMI – LM divided by the square of height – kg/m^2^) were the outcomes. APMT was measured using a skinfold caliper. The mean of three measurements in the non-dominant hand was used in the analyses. APMT was described according to socio-demographic characteristics and nutritional status. The relationship between APMT and both LM and LMI was evaluated by correlation coefficient and linear regression using APMT as a single anthropometric parameter and also in addition to BMI.

**Results:**

APMT was assessed in 3485 participants. APMT was higher in males, non-whites, less-schooled and obese individuals. APMT was moderately correlated to LM and LMI (ranged from 0.44 to 0.57). Correlation coefficients were higher for LMI as outcome and in females (LM: 0.51 and LMI: 0.57). APMT explained 19% and 26% of the variance in LM in males and females, respectively, whereas it explained 26% and 33% of the variance in LMI. APMT increased the prediction for LM in 3 and 4 percentage points in males and females, in comparison to explained by BMI. BMI explained 48% and 59% of the variance of LMI in males and females whereas APMT increased it to 51% and 62% for both sexes, respectively.

**Conclusions:**

Results were not good enough to promote the APMT as a single predictor of LM or LMI in epidemiological studies. APMT has a little predictive capacity in estimating LM or LMI when BMI is also considered.

## 1 Introduction

Nowadays, there is a growing importance of body composition evaluation in several fields [1]. The measurement of body composition allows documenting the efficiency of nutrition support, tailoring the choice of nutritional behaviors and therapies, whereas only body weight does not allow objectively the same approach [1]. Assessment of fat mass has been the main focus of several studies in the last decades due to the importance of the evaluation of the body fat *per se* as well as its corporal distribution [2], [3]. However, lean mass (LM) has also recently attracted major attention in the scientific literature, given its role as an important predictor of clinical outcomes [4], [5]. It has been reported that LM is a fundamental determinant of growth and development [6], as well as an important clinical marker of diseases and aging processes [7].

Several methods to evaluate body compartments have been developed and, subsequently, adapted for use in different scenarios. Devices such as dual energy X-ray absorptiometry (DXA) and air-displacement plethysmography have been proven reliable in epidemiological scenario [8]. Unfortunately, given the high costs, technical complexity and low availability of the methods, their use is restricted in clinical and research environments.

In population-based studies, the availability of simple and minimally invasive methods with lower costs is important. With that in mind, anthropometric measurements have been largely used in epidemiological studies to assess fat mass – such as waist and hip circumference and skinfold thickness [9], [10]. However, the growing attention to LM as a predictor of clinical outcomes highlights the necessity of reliable tools, which can easily assess LM in different cohorts.

Previous studies have reported that low adductor pollicis muscle thickness (APMT) could be used as a proxy of low lean mass in clinical scenario [11], [12], [13]. This muscle has an easily accessible location in the hands and suffers minimal interference of the subcutaneous fat tissue in its thickness' assessment. APMT has been used mainly in the clinical environment, particularly in surgical, renal, long-term hospitalized or critical care patients [11], [14], [15], [16], [17], as a predictor of malnutrition, length of stay and mortality. However, its use in the general healthy population has been scarcely studied.

Few studies have described APMT in healthy subjects according to demographic characteristics. Lameu et al. [13] observed a positive correlation between APMT and arm muscle circumference, arm muscle area and calf circumference, but did not find any meaningful correlations with fat parameters. Gonzalez et al. [18] found a positive correlation of APMT with BMI, but weak correlations with weight, height and age. To our knowledge, no previous study has compared APMT and LM measured by reference methods are inexistent.

The present study aimed to assess the relationship between the APMT and LM among young adults in South Brazil.

## 2 Materials and methods

Data used for this analysis were collected as part of the last follow-up of the 1982 Pelotas Birth Cohort Study. These subjects (*n* = 5914 at birth) were followed-up on several occasions, and further details about this cohort are available elsewhere [19], [20].

From June, 2012 to February, 2013, the cohort members were invited to visit the research clinic, where they were interviewed and examined. All procedures were approved by the Ethics Committee in Research of the Faculty of Medicine at Federal University of Pelotas and a written informed consent was obtained from all subjects.

Subjects were categorized by BMI according to the World Health Organization recommendation [21]. Standing height was measured to the nearest 1 mm, using a wooden stadiometer with the barefooted subjects. Weight was assessed using a pletismography scale (BodPod^®^ – Cosmed, Italy), with the precision of 0.01 kg. Their economic status was also assessed, based on asset index, having a full-time maid and the head of the family's schooling. This allowed us to stratify subjects in wealth groups from A – richest – to E – poorest, according to the Brazilian Research Association Institute criterion.

APMT measurement (mm) was performed using a Lange^®^ skinfold caliper (Beta Technology – Santa Cruz, CA, USA). Measurements were taken as subjects sat upright in a chair with their legs, arms and backs supported. Arms were set at a 90° angle from the elbow using the chairs arm rest. APTM was measured with the skinfold caliper in the vertex of an imaginary triangle formed by the extension of the thumb and the index finger, under the continuous pressure of 10 g/mm. The mean of three measurements was used [18]. The non-dominant APMT was chosen for consideration in this study – therefore, the values obtained from the left hand of right-handed subjects, and from the right hand of the left-handed ones, were used. Examiners were trained and standardized using acceptable technical errors of measurement calculated based on Habicht's publication [22] for all anthropometric measurements. Exclusion criteria for APMT were factors that could influence the execution of daily movements, such as pregnancy; tendinitis; current injuries or deterioration of mobility due to previous injuries or accidents in at least one of the arms or hands; fractures in the upper limbs in the last six months; wheelchair use, mental disorders and degenerative diseases (e.g. fibromyalgia).

LM was assessed using DXA (Lunar Prodigy Advance – GE^®^, Germany). Total body DXA scans were not performed in pregnant women and subjects weighing more than 120 kg or taller than 1.92 m. Subjects with metal surgical implants and irremovable metal items were excluded from examination. Subjects that could not fit in the DXA scan area were submitted to half-body scans of their right side to estimate total body composition. Lean Mass Index (LMI) was also calculated by dividing the LM (kg) by the square of height (m), as proposed by VanItallie [23].

All analyses were stratified by sex. Student's *t*-test or Analysis of Variance (ANOVA) was used in the bivariate analysis. Scatter plots were used to show the relationship between APMT and LM (kg) or LMI (kg/m^2^), and Pearson's correlation was also determined. Regression coefficients and adjusted coefficient of determination (adjusted *R*^2^) were both estimated using linear regression: first, for APMT only; later, using anthropometric variable in addition to BMI. Significance level was set in 5%.

## 3 Results

In 2012–3, 3701 participants from the original 1982 Pelotas Birth Cohort were interviewed. The follow-up rate was 68.1% (including 325 known deaths). After exclusion, 3338 individuals were DXA scanned. APMT was, on average, 24.2 mm (sd = 4.2) and 19.4 mm (sd = 3.9) for males and females, respectively. Table 1 shows that APMT was higher among non-white subjects. Females from the highest economic status presented lower APMT (*p* < 0.001), whereas among males the same relationship was observed but it was not statistically significant. The highest schooling group showed lower APTM than the two lowest groups in both males (*p* < 0.001) and females (*p* < 0.001). Nutritional status was positively associated with APMT (*p* < 0.001).

**Fig. 1:**
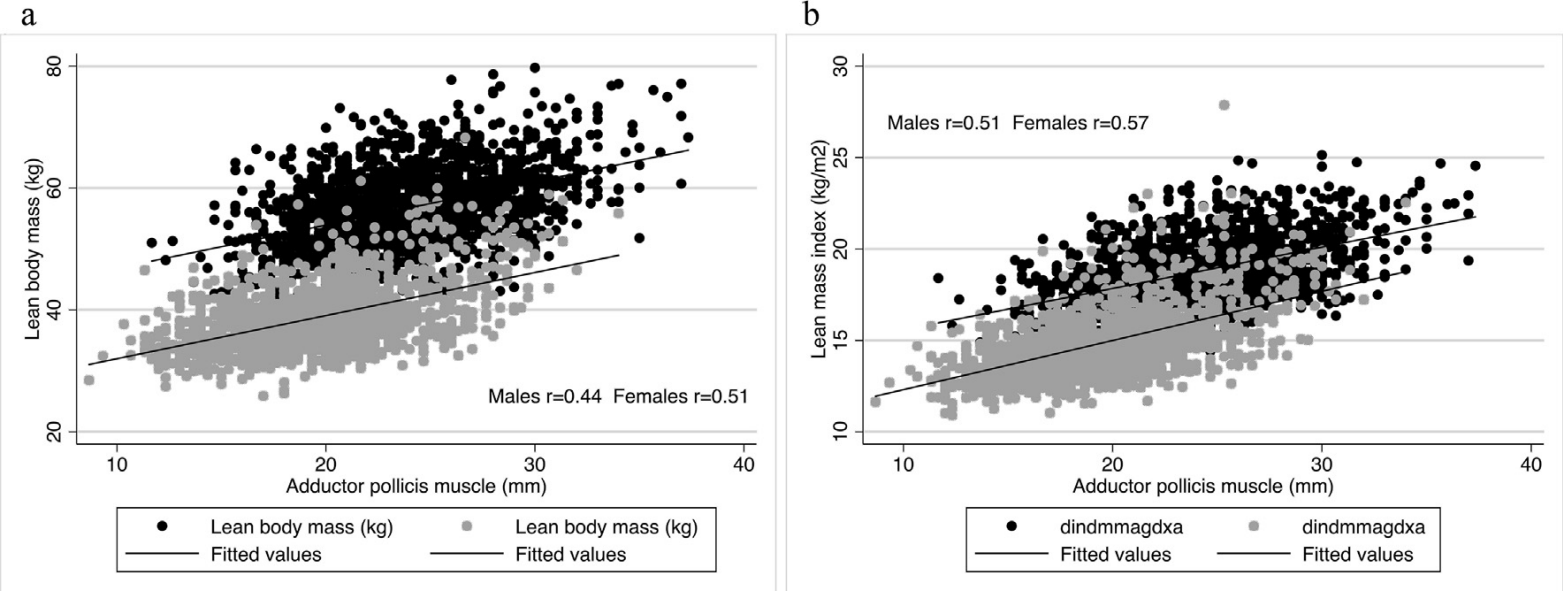
Relationship of adductor pollicis muscle thickness with lean mass and lean mass index by sex in young adults from Southern Brazil. (a) Adductor pollicis muscle thickness in relation to lean mass; (b) adductor pollicis muscle thickness in relation to lean mass index.

Fig. 1 shows that APMT was positively correlated with LM and LMI, regardless of the sex. Pearson's coefficients were higher in females than in males. In females, the correlation between APMT and LM was *r* = 0.51, whereas, in males, *r* = 0.44. For LMI, the correlation coefficient was 0.51 and 0.57, for males and females, respectively.

Regression coefficients of APMT in the LM prediction were similar for males (*β* = 0.71, 95% CI = 0.64; 0.78) and females (*β* = 0.71, 95% CI = 0.65; 0.76), though the coefficient of determination was slightly higher for females (26.3%) than males (19.1%). Coefficient of determination for APMT was higher in the LMI prediction than for the LM prediction. APMT explains 26% and 33% in the variation of LMI in males and females, respectively (Table 2).

**Fig. 2:**
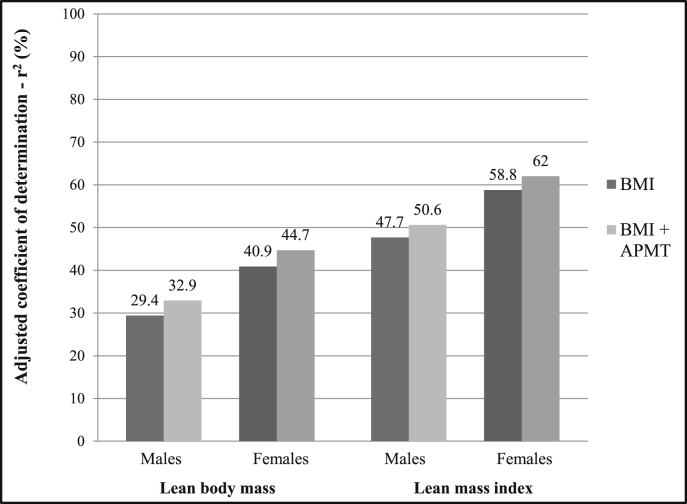
Adjusted coefficients of determination (*r*^2^) of adductor pollicis muscle thickness in the prediction of lean body mass (LBM) and lean mass index (LMI) of young males and females from a Southern Brazilian cohort. BMI – Body mass index; APMT – adductor pollicis muscle thickness.

BMI predicted around 30% and 41% of the LM variation in males and females, respectively (Fig. 2). APMT increased the LM prediction by 3 and 4 percentage points in males and females. BMI explained 48% and 59% of the LMI variation in males and females, whereas APMT increased it to 51% and 62% for both sexes, respectively.

## 4 Discussion

This was the first study that evaluated the relationship between APMT and LM assessed by DXA, an accurate and reliable method in the measurement of body composition compartments. APMT was higher in males, lower in high-educated and richer individuals and was positively related to the nutritional status. Correlation coefficients for the relationship between APMT and both LM and LMI were higher in females. Coefficient of determination of APMT was higher for LMI. APMT alone was able of predicting about 33% of the variation of LMI in females. However, the increase in the prediction of LM or LMI promoted by the APMT when used in conjunction with BMI was low.

Concerning the description of APMT in our young population, APMT values from our study were similar to those found in healthy males and females with approximately the same age described by Gonzalez et al. [18]. However, another Brazilian study found APMT values much lower than our results [13]. Methodological differences from these studies should be considered since Gonzalez et al. [18] reported that lower values found by Lameu et al. [13] can be possibly attributable to measurement errors derived from misplacement of the skinfold caliper from the correct anatomic point. In this case, the lower measurements obtained would be from the skinfold thickness near the muscle, not the APMT. The current study trained and standardized the examiners, filling the existing gap concerning reliable APMT measurements. Still, there are several other studies that evaluated the APMT performance in the clinical scenario, using unhealthy populations. However, due to the subjects' demographic characteristics and, specially, health status, the comparison with our results is unviable.

This study was aimed in assess the prediction of LM by APMT. However, the adductor pollicis muscle was first used to study muscle function through electric stimulation of the ulnar nerve [24]. The use of its thickness as a possible nutritional assessment parameter is recent. Given the method's appliance practicality, portability and low cost, it would be a promissory tool for epidemiological field situations, if it was able to generate an adequate prediction of LM. However, results from the current study were not good enough to encourage the use of APMT in the estimative of LM in large healthy adult populations, mainly because it adds little to the explanation of the total variance in lean mass already promoted by BMI. APMT could be a good predictor of appendicular skeletal muscle mass (ASM), a lean mass measurement from arms and legs that reflects mainly muscle. However, correlation coefficients between APMT and ASM were 0.42 and 0.51 in males and females, respectively (data not shown).

Regarding other LM predictors, there is a large number of anthropometrics measurements used as such. They are generally combined to other anthropometrical assessments, as weight and height, and included in prediction equations with variables such as sex and, sometimes, skin color. Variables such as skinfolds, waist and hip circumferences are usually included as negative predictors of LM in those equations [25], [26], [27], whereas knee height [28], arm [26], [29], calf [26], [29] and thigh circumferences [27], [29] seem to improve the explained variance of those equations, presenting a positive relationship with LM or skeletal muscle mass of adults and elderly. The use of anthropometric-based methods, such as thigh or calf muscle cross-sectional areas and volumes derived from circumference and skinfold thickness measurements, overestimated the same measurement from magnetic resonance imaging [30], [31]. Overestimation of muscle mass by anthropometric measurements was also suggested by several studies included in a recent systematic review from Al-Gindan et al. [25].

It is suggested that APMT is not only influenced by the amount of skeletal muscle mass, but is also influenced by other variables. For example, it is suggested an important influence of the body frame in APMT. Lameu et al. [13] found a progressive increase in the APMT of individuals with a small, medium or large body frame, evaluated by the wrist circumference. In addition, APMT have been previously associated with occupation [18], which requires greater attention, since APMT could be positively biased by the occupation with physical hand effort. On the other hand it could also be a marker of higher levels of occupational physical activity, reflecting higher LM values.

In the previously referred study with healthy individuals from Lameu et al. [13], despite of the possibility of methodological peculiarities already described above, interesting findings must be considered. APMT failed to correlate with triceps skinfold thickness and arm fat area (fat parameters), but had a positive low-to-moderate correlation with calf circumference (*r* = 0.35), arm muscle area (*r* = 0.40) and arm muscle circumference (*r* = 0.42). Correlation coefficients of APMT with LM and LMI in the current study were around 0.50, although increase in explained LM and LMI variance was low when BMI is already considered in the prediction model.

Limitations of this study mainly concern the assessment of a population with the same age, failing to explore variations in the prediction related to the aging process. In addition, the muscle compartment could not be isolated from the LM. This may have biased the results, because APMT reflects mainly the muscle measurement, with low interference from the body water compartment that is also included in the total lean mass. On the other hand, the current study was able to fill the existing knowledge gap concerning the comparison between APMT and whole-body LM evaluated by a reliable method such as DXA, Another strong point of the study was the concern with adequate training and standardization of examiners. Finally, the peak of physical capacity (muscle and bone strength and mass) is reached up to the end of the third decade of life [32].

In summary, APMT was moderately positively correlated with LM and LMI. The performance of APMT in predicting LM was better if height was taken into account (LMI), and in females. However, increases in the coefficient of determination promoted by APMT were low when BMI is already considered. Based on these results, APMT was not considered a good predictor for LM in a generally healthy adult population.

## Statement of authorship

RMB performed the statistical analyses and conceived the study. RMB, BLH, SPO, TGBS, MCG, MCA and DPG drafted the manuscript. TGBS and MCG performed the literature review. SPO performed the training of the anthropometrists. BLH and DPG were the principal investigators of last follow-up of 1982 Pelotas Birth Cohort. All authors revised and approved the final version of the manuscript.

## Conflict of interest

The authors declare that they have no conflict of interest.

## Funding sources

The 1982 birth cohort study was supported by the Wellcome Trust Initiative entitled Major Awards for Latin America on Health Consequences of Population Change, grant entitled: “Implications of early life and contemporary exposures on body composition, human capital, mental health and precursors of complex chronic diseases in three Brazilian cohorts (1982, 1993 and 2004)”. Previous phases of the study were supported by the International Development Research Center, The World Health Organization, Overseas Development Administration, European Union, National Support Program for Centers of Excellence (PRONEX), the Brazilian National Research Council (CNPq) and Brazilian Ministry of Health.

## Figures and Tables

**Table 1:** Adductor pollicis muscle thickness (mm) according to socio-demographic characteristics and nutritional status in young adults from Pelotas, Brazil.

	APMT (mm)					
Males			Females		
*n*	Mean (sd)	*p*	*n*	Mean (sd)	*p*
Skin color			0.006			<0.001
White	1296	24.0 (4.1)		1341	19.1 (3.8)	
Non-white	438	24.6 (4.3)		408	20.6 (3.9)	

Economic status			0.054			<0.001
A/B (richest)	939	24.1 (4.2)		856	18.9 (3.8)	
C	395	24.7 (4.2)		425	20.7 (3.8)	
D/E (poorest)	41	25.1 (4.1)		61	20.3 (4.5)	

Schooling (years)			<0.001			<0.001
0–8	489	25.0 (4.2)		396	20.5 (3.7)	
9–11	548	24.7 (4.2)		490	20.5 (3.8)	
≥12	669	23.2 (4.0)		841	18.4 (3.7)	

Nutritional status (BMI)			<0.001^a^			<0.001^a^
<18.5	24	18.8 (2.8)		44	15.7 (3.1)	
18.5–24.9	615	22.0 (3.6)		789	17.6 (3.1)	
25.0–29.9	702	25.5 (3.5)		497	19.9 (2.9)	
≥30	380	27.4 (4.0)		411	22.9 (3.8)	

APMT – adductor pollicis muscle thickness.

Economic status according to Brazilian Research Association Institute criterion.

a Linear trend test.

**Table 2:** Linear regression coefficients of prediction of lean body mass and lean mass index by adductor pollicis muscle thickness in young adults from Pelotas, Brazil.

	Lean body mass (kg)			Lean mass index (kg/m^2^)		
Coefficient (95% CI)	*p*	Adj *R*^2^	Coefficient (95% CI)	*p*	Adj *R*^2^
*Males*						
APMT (mm)		<0.001	0.191		<0.001	0.259
*α*	39.74 (37.99; 41.49)			13.31 (12.85; 13.77)		
*β*	0.71 (0.64; 0.78)			0.23 (0.21; 0.25)		

*Females*						
APMT (mm)		<0.001	0.263		<0.001	0.325
*α*	24.90 (23.78; 26.02)			9.61 (9.25; 9.98)		
*β*	0.71 (0.65; 0.76)			0.27 (0.25; 0.29)		

APMT – adductor pollicis muscle thickness.
